# Supplementary figures and images for: Routes of Albumin Overload Toxicity in Renal Tubular Epithelial Cells
Source: Int J Mol Sci. 2023 Jun 1;24(11):9640. doi: 10.3390/ijms24119640 (PMC10253691; doi:10.3390/ijms24119640)

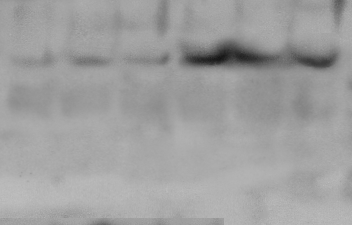

Supplement: Supplementary file 1 [file ijms-24-09640-s001.zip › 4-HNE 3rd exp.tif]

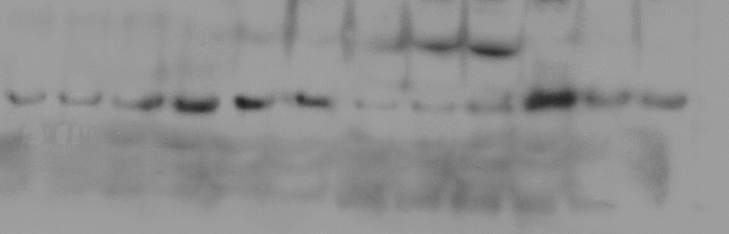

Supplement: Supplementary file 1 [file ijms-24-09640-s001.zip › 4-HNE.tif]

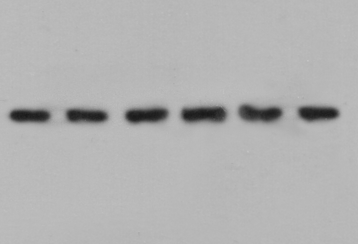

Supplement: Supplementary file 1 [file ijms-24-09640-s001.zip › Actin 1 3rd exp.tif]

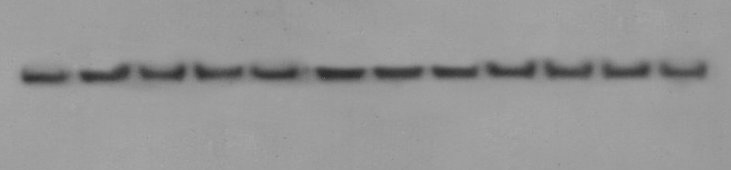

Supplement: Supplementary file 1 [file ijms-24-09640-s001.zip › Actin 1 BSA and NUC.tif]

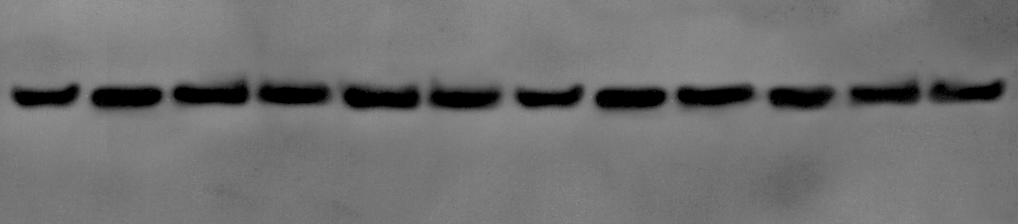

Supplement: Supplementary file 1 [file ijms-24-09640-s001.zip › Actin 1.tif]

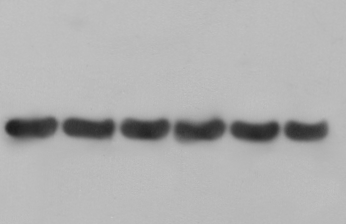

Supplement: Supplementary file 1 [file ijms-24-09640-s001.zip › Actin 2 3rd exp.tif]

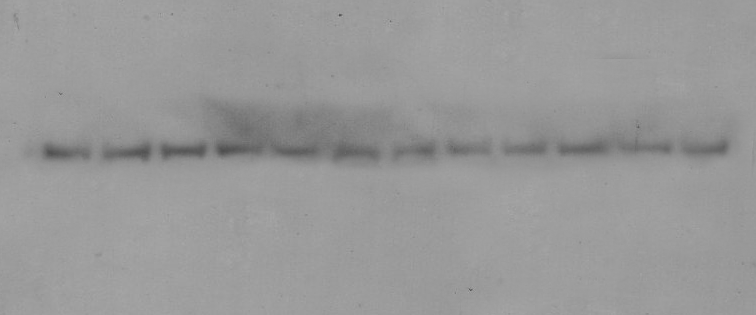

Supplement: Supplementary file 1 [file ijms-24-09640-s001.zip › Actin 2 BSA and NUC.tif]

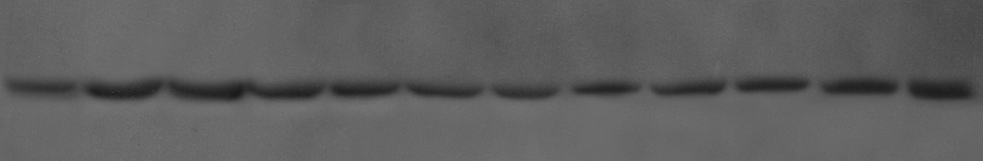

Supplement: Supplementary file 1 [file ijms-24-09640-s001.zip › Actin 2.tif]

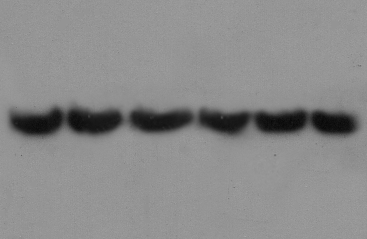

Supplement: Supplementary file 1 [file ijms-24-09640-s001.zip › Actin 3 3rd exp.tif]

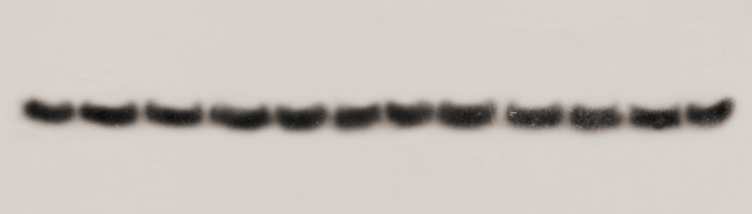

Supplement: Supplementary file 1 [file ijms-24-09640-s001.zip › ACTIN 3.tif]

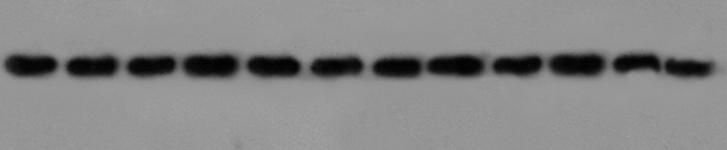

Supplement: Supplementary file 1 [file ijms-24-09640-s001.zip › ACTIN 4.tif]

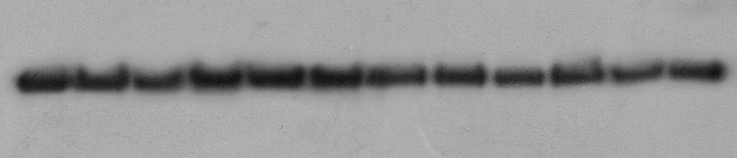

Supplement: Supplementary file 1 [file ijms-24-09640-s001.zip › ACTIN 5.tif]

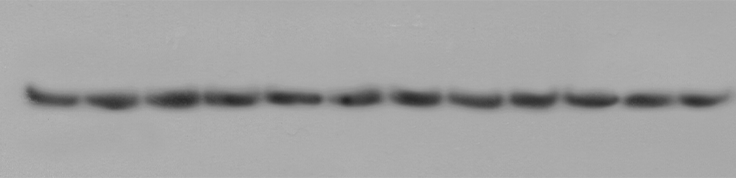

Supplement: Supplementary file 1 [file ijms-24-09640-s001.zip › ACTIN 6.tif]

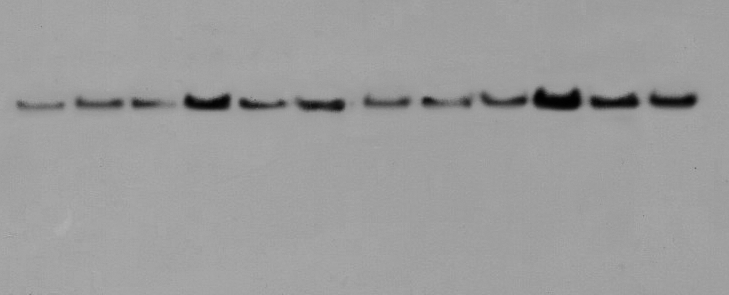

Supplement: Supplementary file 1 [file ijms-24-09640-s001.zip › ATF-3.tif]

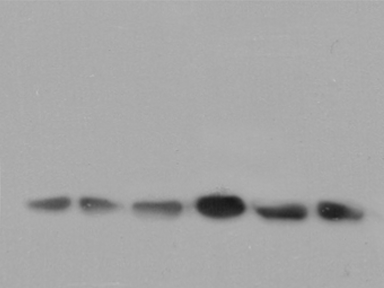

Supplement: Supplementary file 1 [file ijms-24-09640-s001.zip › ATF3 3rd exp.tif]

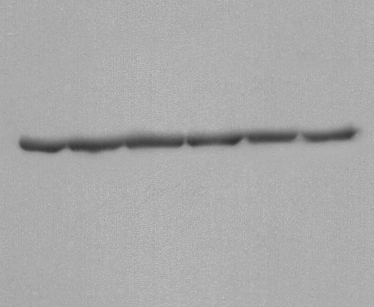

Supplement: Supplementary file 1 [file ijms-24-09640-s001.zip › ATM 3rd exp.tif]

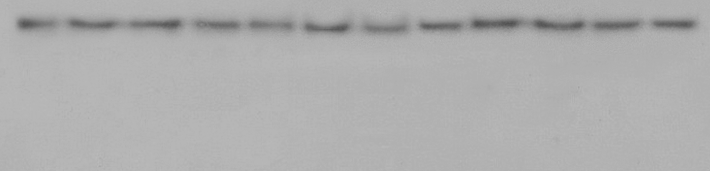

Supplement: Supplementary file 1 [file ijms-24-09640-s001.zip › ATM.tif]

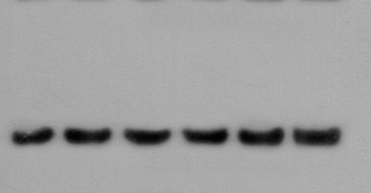

Supplement: Supplementary file 1 [file ijms-24-09640-s001.zip › Bax 3rd exp.tif]

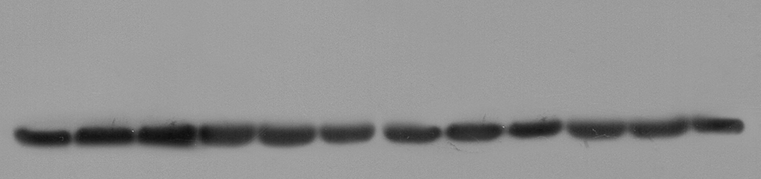

Supplement: Supplementary file 1 [file ijms-24-09640-s001.zip › Bax.tif]

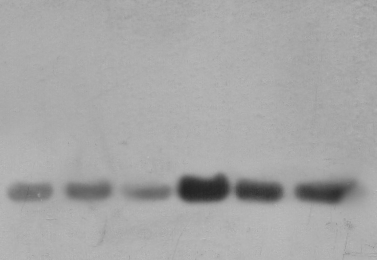

Supplement: Supplementary file 1 [file ijms-24-09640-s001.zip › CC-3 3rd exp.tif]

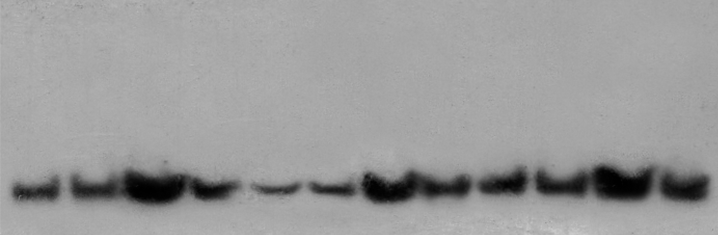

Supplement: Supplementary file 1 [file ijms-24-09640-s001.zip › CC-3 BSA and NUC.tif]

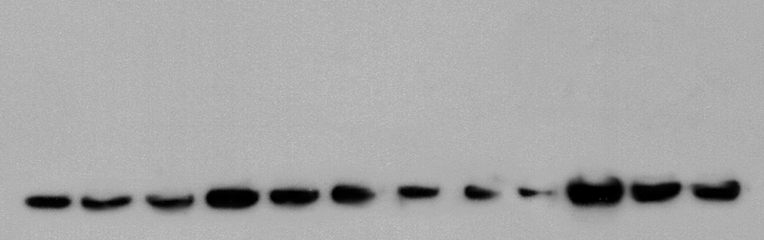

Supplement: Supplementary file 1 [file ijms-24-09640-s001.zip › CC-3.tif]

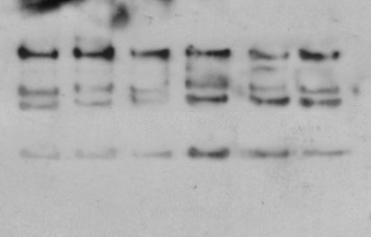

Supplement: Supplementary file 1 [file ijms-24-09640-s001.zip › CC-8 3rd exp.tif]

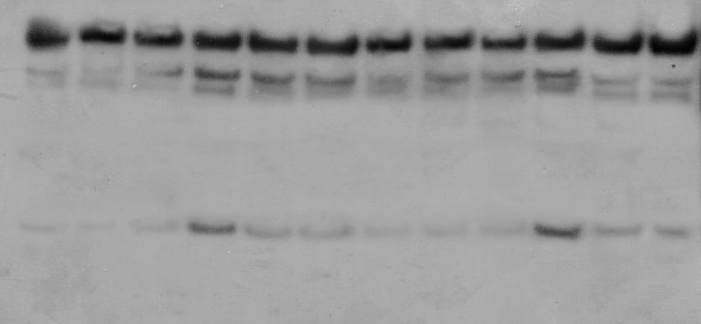

Supplement: Supplementary file 1 [file ijms-24-09640-s001.zip › CC-8.tif]

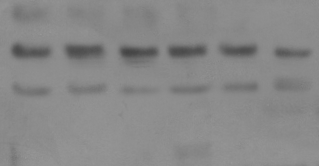

Supplement: Supplementary file 1 [file ijms-24-09640-s001.zip › CC-9 3rd exp.tif]

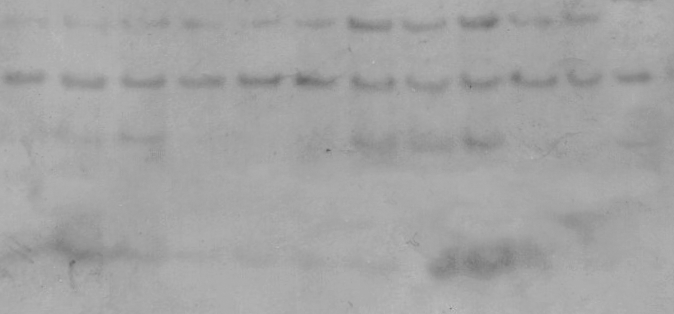

Supplement: Supplementary file 1 [file ijms-24-09640-s001.zip › CC-9.tif]

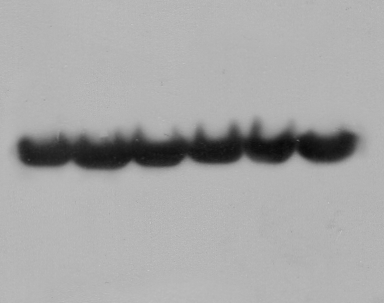

Supplement: Supplementary file 1 [file ijms-24-09640-s001.zip › CHOP 3rd exp.tif]

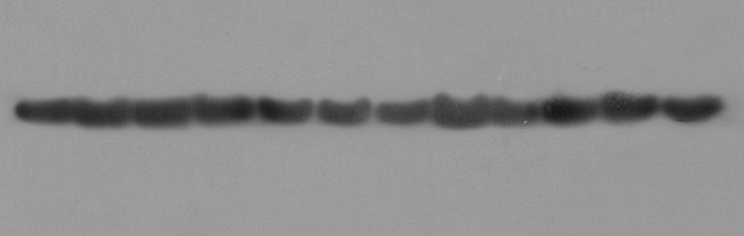

Supplement: Supplementary file 1 [file ijms-24-09640-s001.zip › CHOP.tif]

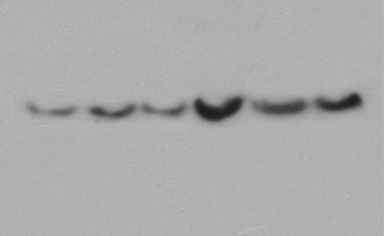

Supplement: Supplementary file 1 [file ijms-24-09640-s001.zip › DR5 3rd exp.tif]

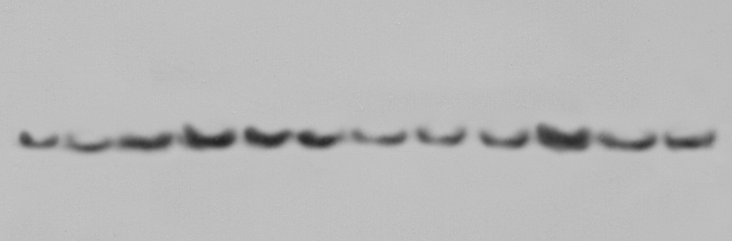

Supplement: Supplementary file 1 [file ijms-24-09640-s001.zip › DR5.tif]

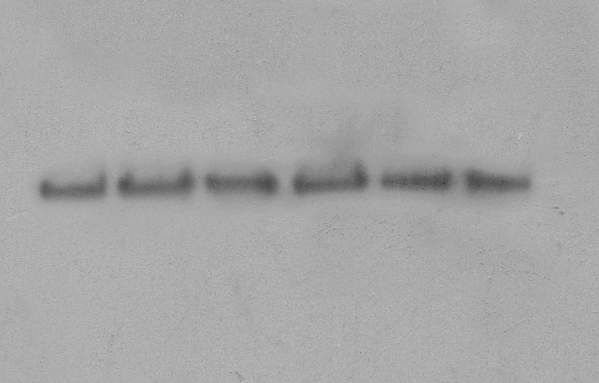

Supplement: Supplementary file 1 [file ijms-24-09640-s001.zip › eIF2╬▒ 3rd exp.tif]

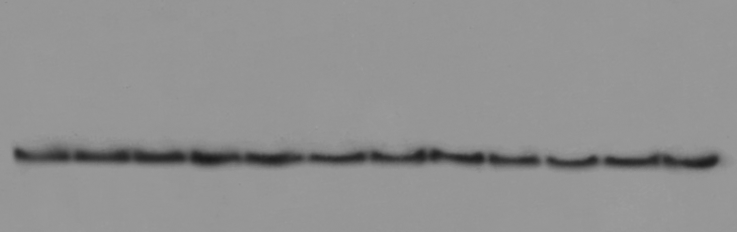

Supplement: Supplementary file 1 [file ijms-24-09640-s001.zip › eIF2╬▒.tif]

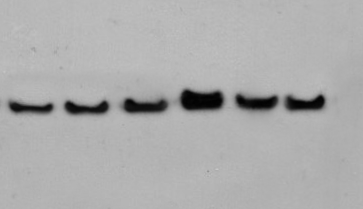

Supplement: Supplementary file 1 [file ijms-24-09640-s001.zip › GLB-1 3rd exp.tif]

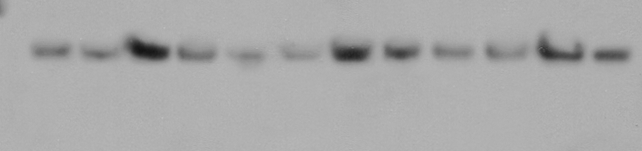

Supplement: Supplementary file 1 [file ijms-24-09640-s001.zip › GLB-1 BSA and NUC.tif]

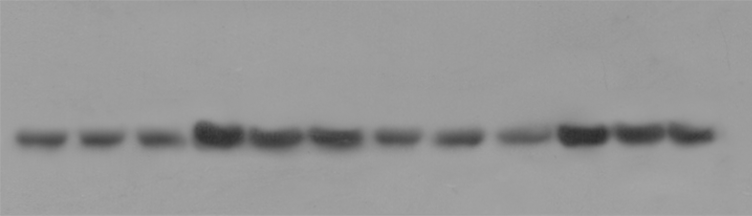

Supplement: Supplementary file 1 [file ijms-24-09640-s001.zip › GLB-1.tif]

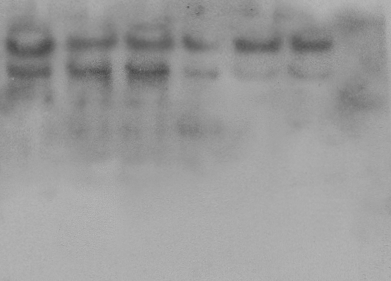

Supplement: Supplementary file 1 [file ijms-24-09640-s001.zip › Ki-67 3rd exp.tif]

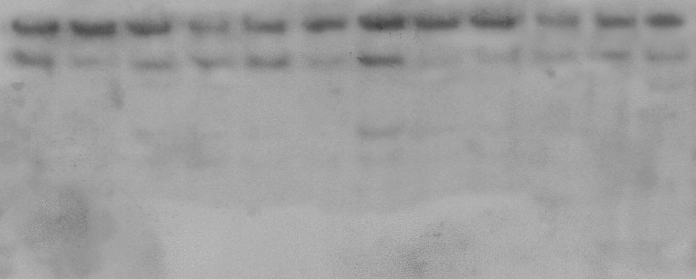

Supplement: Supplementary file 1 [file ijms-24-09640-s001.zip › Ki-67.tif]

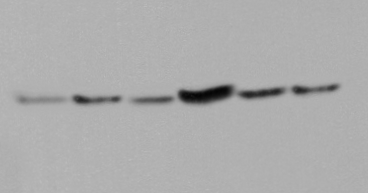

Supplement: Supplementary file 1 [file ijms-24-09640-s001.zip › p-ATM 3rd exp.tif]

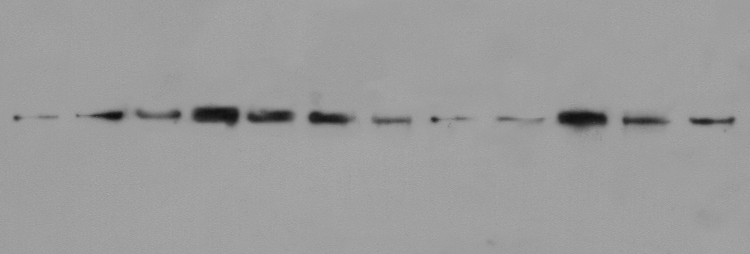

Supplement: Supplementary file 1 [file ijms-24-09640-s001.zip › p-ATM.tif]

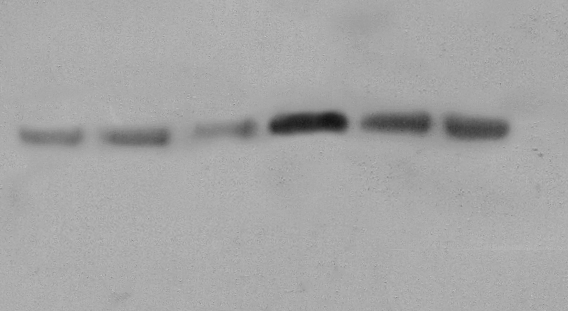

Supplement: Supplementary file 1 [file ijms-24-09640-s001.zip › p-eIF2╬▒ 3rd exp.tif]

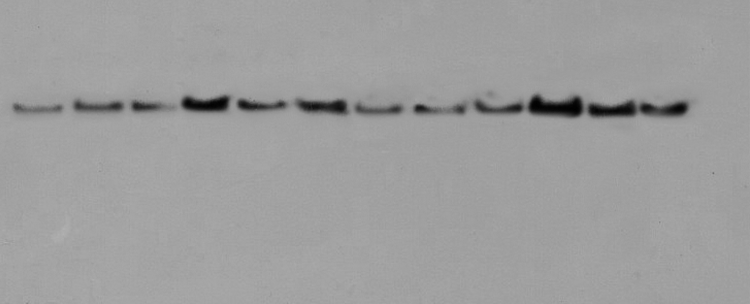

Supplement: Supplementary file 1 [file ijms-24-09640-s001.zip › p-eIF2╬▒.tif]

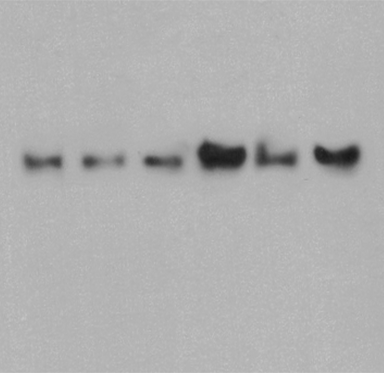

Supplement: Supplementary file 1 [file ijms-24-09640-s001.zip › p-p53 3rd exp.tif]

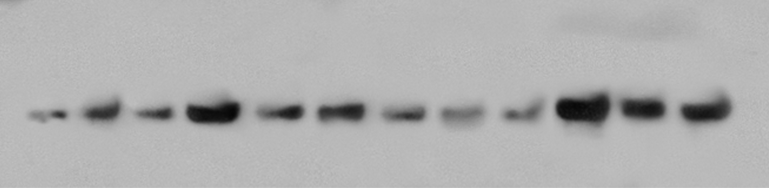

Supplement: Supplementary file 1 [file ijms-24-09640-s001.zip › p-p53.tif]

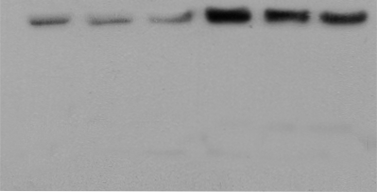

Supplement: Supplementary file 1 [file ijms-24-09640-s001.zip › p-PERK 3rd exp.tif]

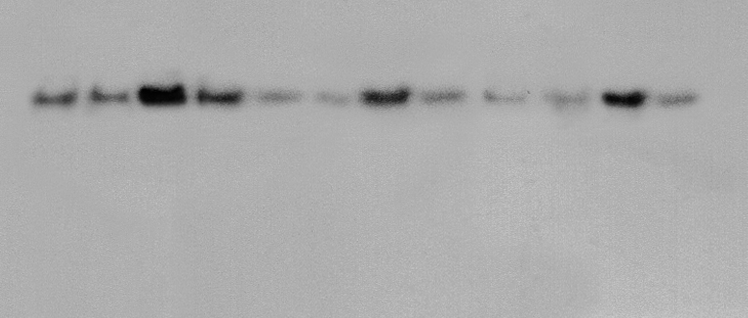

Supplement: Supplementary file 1 [file ijms-24-09640-s001.zip › p-PERK BSA and NUC.tif]

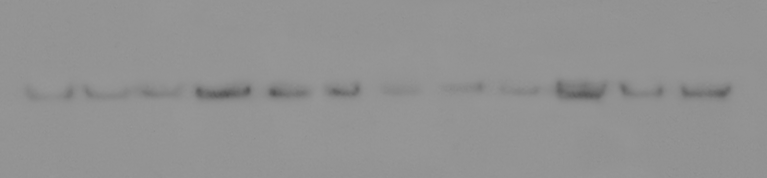

Supplement: Supplementary file 1 [file ijms-24-09640-s001.zip › p-PERK.tif]

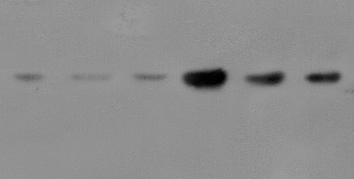

Supplement: Supplementary file 1 [file ijms-24-09640-s001.zip › p16 3rd exp.tif]

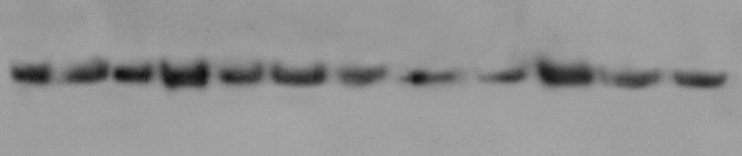

Supplement: Supplementary file 1 [file ijms-24-09640-s001.zip › p16.tif]

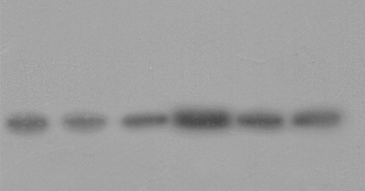

Supplement: Supplementary file 1 [file ijms-24-09640-s001.zip › p21 3rd exp.tif]

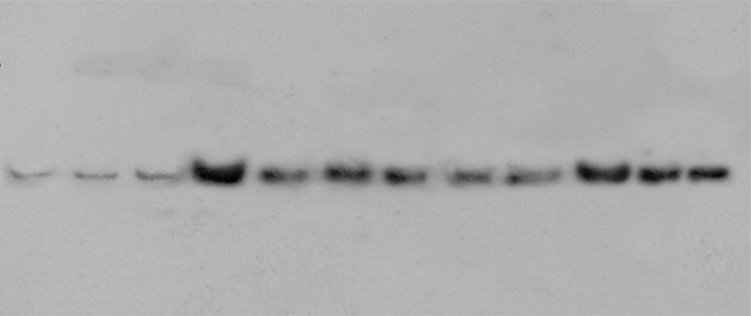

Supplement: Supplementary file 1 [file ijms-24-09640-s001.zip › p21.tif]

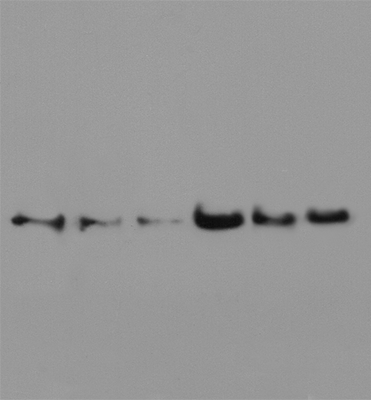

Supplement: Supplementary file 1 [file ijms-24-09640-s001.zip › p53 3rd exp.tif]

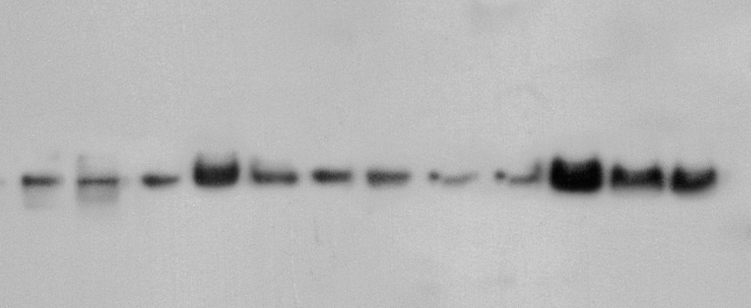

Supplement: Supplementary file 1 [file ijms-24-09640-s001.zip › p53.tif]

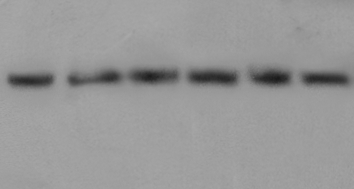

Supplement: Supplementary file 1 [file ijms-24-09640-s001.zip › PERK 3rd exp.tif]

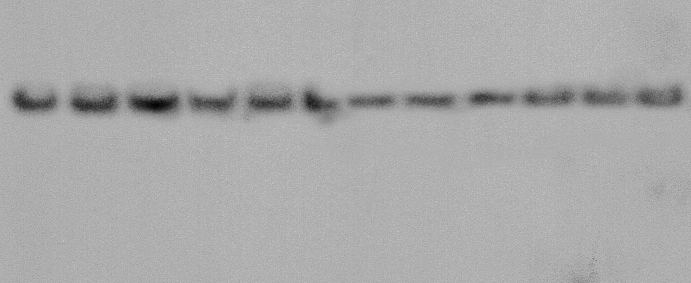

Supplement: Supplementary file 1 [file ijms-24-09640-s001.zip › PERK BSA and NUC.tif]

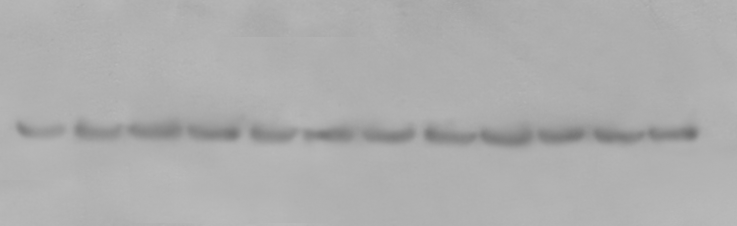

Supplement: Supplementary file 1 [file ijms-24-09640-s001.zip › PERK.tif]

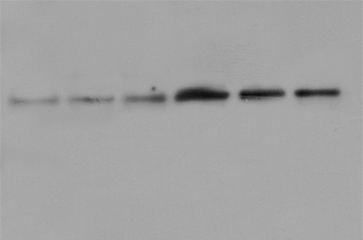

Supplement: Supplementary file 1 [file ijms-24-09640-s001.zip › ╬▒-SMA 3rd exp.tif]

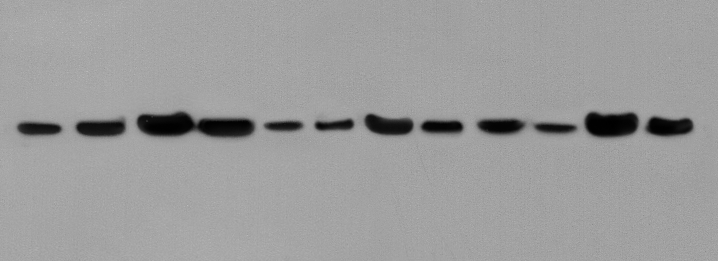

Supplement: Supplementary file 1 [file ijms-24-09640-s001.zip › ╬▒-SMA BSA and NUC.tif]

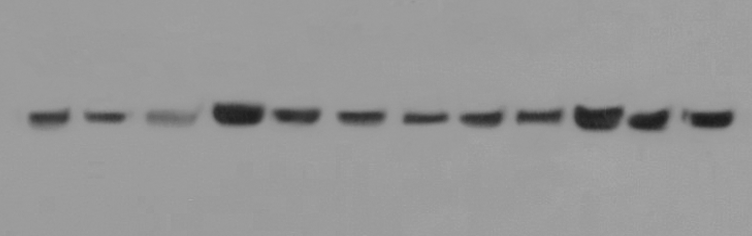

Supplement: Supplementary file 1 [file ijms-24-09640-s001.zip › ╬▒-SMA.tif]

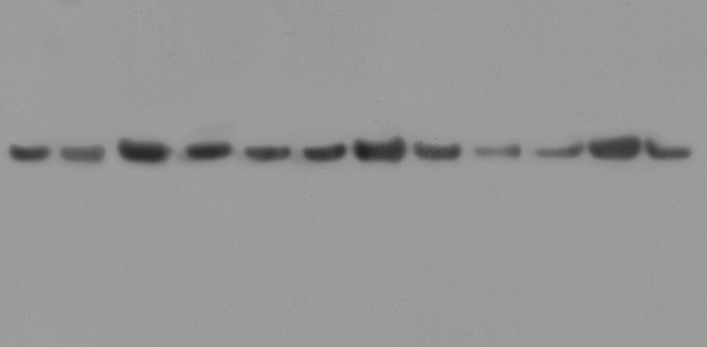

Supplement: Supplementary file 1 [file ijms-24-09640-s001.zip › ╬│-H2AX BSA and NUC.tif]

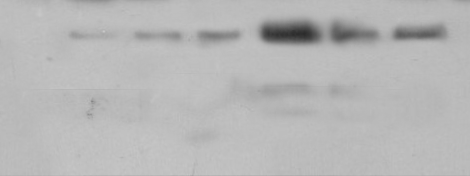

Supplement: Supplementary file 1 [file ijms-24-09640-s001.zip › ╬│-╬ù2╬æ╬o 3rd exp.tif]

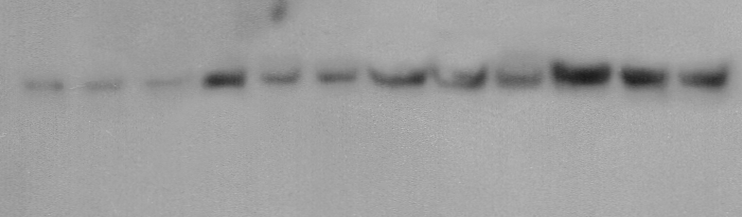

Supplement: Supplementary file 1 [file ijms-24-09640-s001.zip › ╬│-╬ù2╬æ╬o.tif]
